# Steady State: A Behavior-Based Educational Game for Time Management

**DOI:** 10.3390/bs16081290

**Published:** 2026-07-28

**Authors:** Francesco Di Nocera, Sara Tinella

**Affiliations:** Department of Planning, Design, and Technology of Architecture, Sapienza University of Rome, 00196 Rome, Italy

**Keywords:** behavior analysis, self-management, self-regulation, time management, procrastination, game-based learning, contingency management, educational game, proof-of-concept, single-case design

## Abstract

This paper introduces Steady State, a noncommercial, physical, two-player educational board game designed to model behavioral processes involved in time management from a behavior-analytic perspective. Although time management has been widely discussed in psychological and educational contexts, operationalized models are still needed to examine task allocation, postponement, and self-management under controlled conditions. The game models self-management under limited resources by arranging explicit response-consequence relations involving energy and stress tokens, task demands, reinforcement contingent on task completion, and weekly differential reinforcement for balanced engagement across domains. It also embeds self-monitoring prompts. A demonstrative pilot using an alternating treatments design (12 sessions with one dyad) compared the full contingency-based game (Condition A) with a procedural comparison condition in which the main response-consequence relations were minimized and stochastic outcomes were emphasized (Condition B). Embedded performance indices, including task postponements, energy remaining at the end of the session, stress accumulation, and planning time, varied more clearly with individual strategy in Condition A, whereas patterns in Condition B were more strongly constrained by stochastic outcomes. We discuss limitations and priorities for more controlled replication. The pilot was designed not to test educational effectiveness or transfer to everyday time management, but to determine whether the game generates measurable and behaviorally interpretable in-game variability.

## 1. Introduction

Managing large amounts of information, meeting deadlines, organizing personal activities, and maintaining productivity require substantial allocation of time. Interest in what is commonly called time management (TM) became prominent in the 1960s and 1970s, driven by practitioners such as Drucker (1967) and Lakein (1973), who introduced techniques for improving work performance, including task lists and structured activities. Earlier, McCay (1959) had outlined a time-management training approach emphasizing analysis of time use, daily planning, task prioritization, and responses to unexpected demands. Many methods have since been developed to improve time-management skills. One of the most influential is Allen’s (2001) Getting Things Done (GTD) method, a task-management system organized around five steps: capture, clarify, organize, reflect, and engage. The method seeks to reduce “mental clutter” by externalizing tasks so that users can focus on execution rather than retention. The popularity of GTD has also influenced the development of time-management software, much of which is explicitly designed around its principles.

From a functional-analytic standpoint, TM can be conceptualized as behavior occurring within defined temporal contexts and shaped and maintained by specific reinforcement contingencies. Definitions in the psychological literature are more or less consistent with this perspective because they emphasize behavior directed toward completing tasks and achieving goals (Adams & Jex, 1997; Aeon et al., 2021; Bond & Feather, 1988; Claessens et al., 2007; Jex & Elacqua, 1999). Across these definitions, three common elements emerge: (1) task-completion behavior, (2) occurrence within explicitly defined temporal intervals, and (3) the use of planning, organizing, and prioritizing strategies to establish contingencies that support desired outcomes (Liu et al., 2009).

Importantly, from a behavior-analytic perspective, the term “time management” can be misleading because time itself is not managed. Rather, behavior occurring within temporal intervals is controlled and modified by reinforcement contingencies. Consequently, terms such as “personal productivity,” “task management,” or “self-management,” which clearly reference observable and measurable behaviors, interventions, resources, and assessment tools, may be more appropriate (Johnston & Pennypacker, 1993; Kazdin, 2001; Skinner, 1953). Nonetheless, given its widespread usage in the literature, we retain “time management” as shorthand for this functional, behavior-analytic conceptualization.

This functional interpretation is consistent with recent proposals to analyze academic procrastination through functional analysis. Svartdal and Løkke (2022) argued that procrastinatory behavior should be examined in terms of antecedent conditions, the specific behaviors involved in delaying goal-relevant activity, and the immediate consequences that may maintain those behaviors. From this perspective, procrastination is not merely a failure of intention or a trait-like deficit, but a pattern of behavior controlled by idiosyncratic antecedent–behavior–consequence relations. This view directly supports the present approach, which treats time-management difficulties as modifiable patterns of task allocation, delay, avoidance, and self-management under specific contingencies.

Initially, interventions described in the literature were predominantly prescriptive, consisting of “how-to” manuals and training programs designed to improve personal productivity through explicit instruction and feedback. More recent analyses (e.g., Claessens et al., 2007) have noted a gap in empirical research, particularly regarding the measurement and operational definition of TM behaviors and the formulation of coherent theoretical models explaining the functional relationships between these behaviors and their outcomes. The lack of a unified operational definition limits the interpretability and generalizability of findings.

Taken together, the available literature suggests that time-management and procrastination are increasingly being interpreted as patterns of behavior shaped by antecedents and consequences rather than as stable traits. However, three areas remain relatively underexplored. First, few studies provide operationalized environments in which planning, task allocation, postponement, and resource management can be observed directly as ongoing behavior. Second, most existing interventions focus on instruction or self-report measures rather than on repeated contact with explicit reinforcement contingencies. Third, there is limited translational research using ecologically meaningful but experimentally controllable tasks that allow session-by-session analysis of how individuals adapt to changing demands and consequences. These gaps motivated the development of Steady State and guided the proof-of-concept evaluation presented in this paper.

The use of board games for educational purposes, including games explicitly designed to embody and communicate abstract social relations, is not new. At the beginning of the twentieth century, Lizzie Magie created The Landlord’s Game to illustrate how monopolies and land rents could burden an economy and how land-value taxation might redistribute those costs. The game circulated in homemade variants and eventually provided the basis for the commercial game Monopoly, although its original critical purpose was effectively reversed: rather than illustrating the social costs of monopoly, the later game rewarded players for creating one (Pilon, 2015). This history illustrates how game mechanics can operationalize complex functional relations, allowing players to encounter their consequences directly rather than learning about them only through verbal instruction. Steady State applies the same general design principle to patterns of planning, task allocation, postponement, and resource management.

The present study addresses these gaps by applying a behavior-analytic framework (Rasmussen et al., 2023) to the design and evaluation of Steady State, a noncommercial educational board game built around contingencies of reinforcement and punishment, including negative reinforcement and escape where relevant, variability in task demands, and self-monitoring components. The game is distributed under CC BY-NC-ND 4.0, and the printable materials are freely available online (Di Nocera & Tinella, 2026). Rather than treating TM as a trait or ability, Steady State arranges explicit antecedents and consequences to shape and maintain adaptive patterns of task engagement across repeated interactions, consistent with functional-analytic accounts of procrastination and self-management.

Rachlin’s research program on self-control (Rachlin & Green, 1972; Rachlin, 1974, 1995) frames procrastination as a choice pattern in which immediate, smaller-sooner relief from deferring effort competes with larger-later outcomes. Commitment responses can stabilize preferences and counter such reversals, for example by precommitting to earlier task initiation and linking it to valued consequences.

Time-stamped behavioral data from e-learning platforms show that task completion often clusters near deadlines, producing group response profiles reminiscent of fixed-interval scalloping and well described by hyperbolic models associated with delay-discounting parameters (Sokolowski & Tonneau, 2021). Complementary evidence from an authentic academic writing task indicates that higher self-reported procrastination is associated with an end-loaded productivity curve, with time on task and keystrokes concentrated near the deadline. Lower procrastination, by contrast, is characterized by steadier work routines. These differences appear more closely related to weaker task-oriented coping than to simple task avoidance (Di Nocera et al., 2023).

The game proposed here serves as a laboratory analog of everyday choice under limited resources and delayed outcomes, a common translational strategy for studying self-regulation with ecologically relevant yet experimentally controllable stimuli and contingencies (e.g., Ecott & Critchfield, 2004).

Accordingly, Steady State provides an operationalized framework for studying time-management behavior through manipulable antecedent-consequence relations, structured task demands, and self-monitoring.

The present study addressed two objectives:

O1: Technological-description objective. To provide a detailed behavior-analytic description of Steady State and show how its mechanics operationalize planning, task allocation, postponement, stress accumulation, and resource management as observable in-game behaviors.
O2: Proof-of-concept objective. To examine, through a demonstrative pilot, whether the game generates measurable and behaviorally interpretable session-by-session variability under a contingency-based arrangement and a stochastic procedural comparison condition.

The pilot evaluation associated with O2 was guided by the following exploratory research questions:

RQ1: Do the embedded in-game measures capture observable and behaviorally interpretable differences in planning, allocation, postponement, and resource-management patterns across repeated sessions?
RQ2: Do performance patterns under Condition A show greater between-player differentiation and a closer descriptive relation to individual planning and allocation choices than patterns under Condition B?

These research questions were exploratory and were not intended to test educational effectiveness or transfer to everyday time management.

## 2. Behavior-Based Design of the Game Mechanics

Steady State is a two-player board game designed to support practice and reflection on self-management under limited resources, competing task demands, and programmed consequences. The name Steady State refers to the behavior-analytic concept of steady-state responding, in which performance becomes relatively stable after initial transients and the remaining variability is bounded and informative. From a functional behavior-analytic perspective, the game is an engineered environment that simulates everyday contingencies related to time management and task completion. It can therefore be conceptualized as a structured contingency-management system that arranges antecedents, responses, and consequences to model and shape adaptive repertoires.

Discriminative stimuli (SDs) include the days on the calendar, task strips, and cards drawn, which signal the availability of reinforcement for certain behaviors.

Operant behaviors include task allocation, decision-making under energy constraints, delegation, and stress mitigation, all of which are selected by their consequences.

Programmed consequences include energy units arranged to function as putative conditioned reinforcers. These contingencies model real-world reinforcement for completion, balance, and proactive planning. Aversive stimulation is introduced through stress units and delegation mechanics. As stress units accumulate, the energy cost of completing tasks increases, thereby modeling elevated response effort as an aversive condition.

The variable-ratio-like arrangement of energy-card delivery is intended to support task persistence by approximating the effects of intermittent reinforcement in everyday productivity contexts.

The review phase incorporates self-evaluation and rule-governed behavior that may influence future responding through self-generated verbal rules.

Overall, the game simulates a contingency system in which limited resources, competing demands, and delayed outcomes create a realistic context for practicing and reflecting on self-management skills.

For clarity, although the game includes energy and stress units, represented by tokens that players can accumulate and spend, these units do not constitute a token economy. They are not generalized conditioned reinforcers exchanged for backup reinforcers during programmed exchange periods, and the game includes no exchange-production schedule.

Table 1 summarizes the correspondence between each game mechanic, its intended behavior-analytic function, and the associated educational objectives.

## 3. Game Description

**Figure 1:**
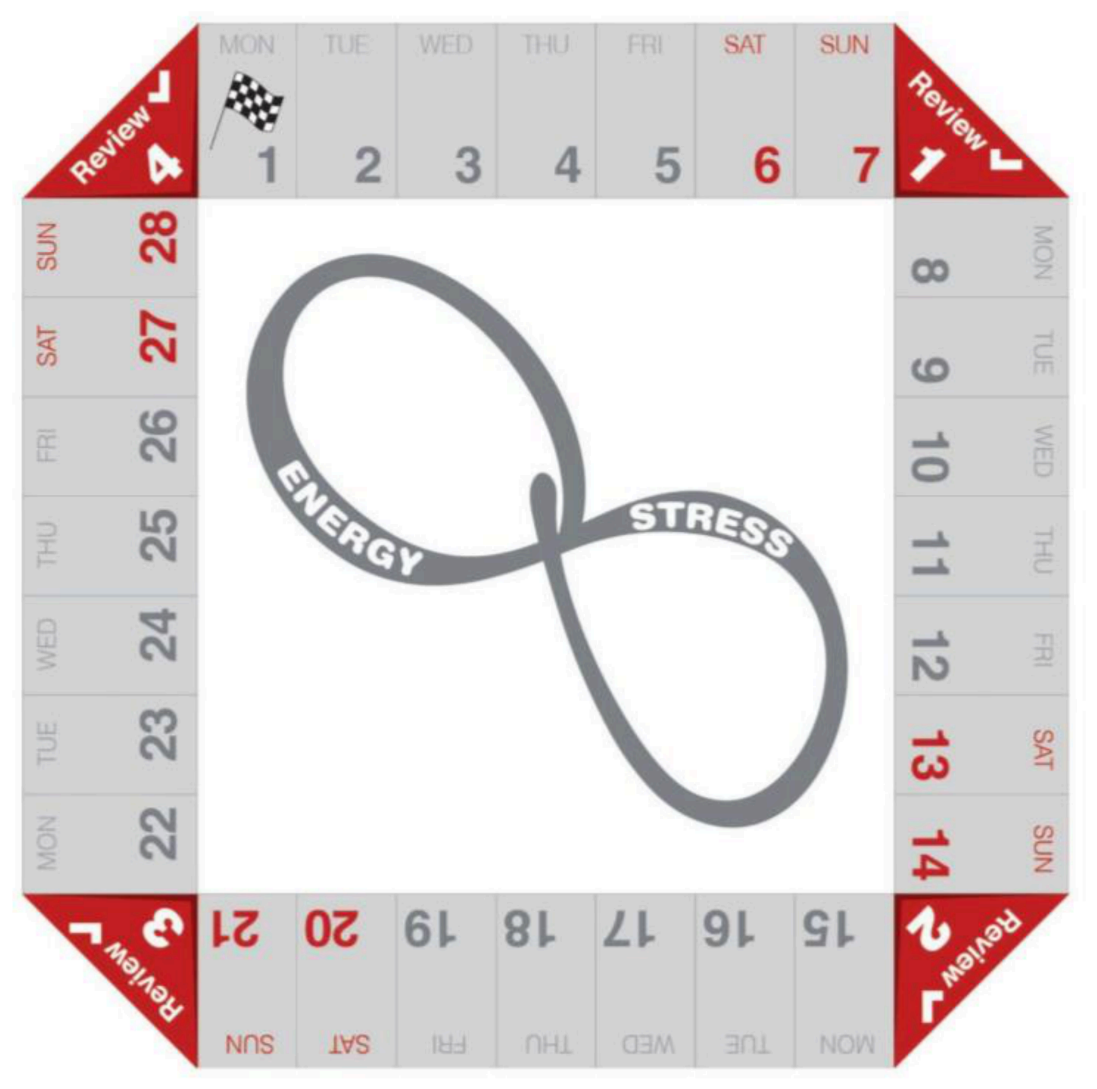
Steady State game board.

The game unfolds over 32 spaces representing the days of four weeks, Monday through Sunday, plus a corner space at the end of each week for the weekly review. Play proceeds around the four sides of a square board, as in Monopoly (Figure 1).

**Figure 2:**
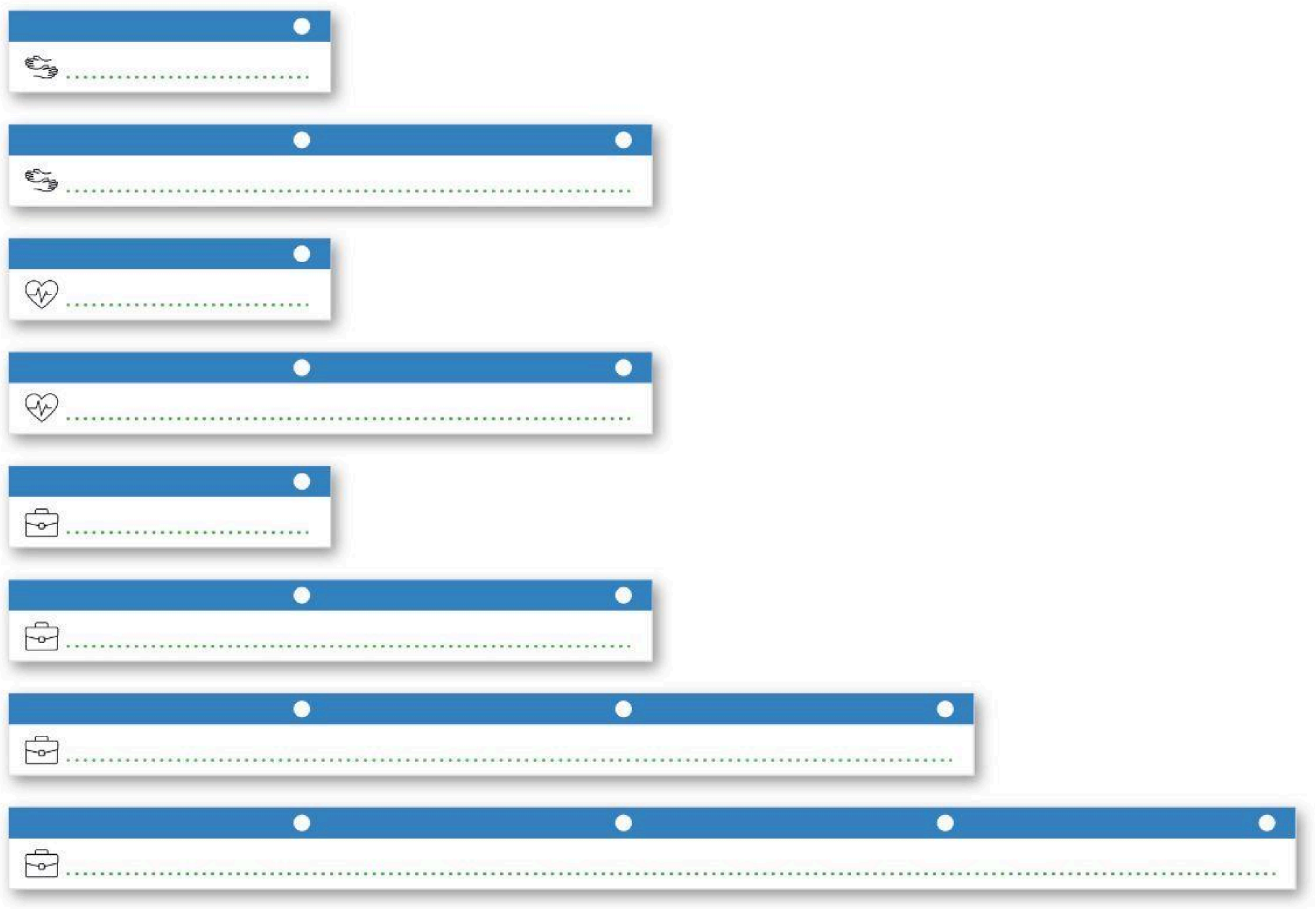
Paper “strips” representing tasks to be completed in each category.

At the beginning of each session, players receive paper task strips (Figure 2). Strip length ranges from one to four spaces, corresponding to days, and each player uses a different color. Tasks are also divided into categories. Work is intentionally overrepresented, with 30 task-days, compared with 9 task-days for Health/Wellbeing and 9 for Relationships/Leisure, to approximate everyday constraints. Specifically:

- Work: 3 strips of 1 day, 3 of 2 days, 3 of 3 days, 3 of 4 days;
- Health/Wellbeing: 3 strips of 1 day, 3 of 2 days;
- Relationships/Leisure: 3 strips of 1 day, 3 of 2 days.

Because the available spaces do not permit all tasks to be allocated without overlap, players must decide which tasks to complete and which to postpone. Postponement can have an immediate reinforcing function because it temporarily reduces response effort and preserves energy units for other tasks. In this sense, it may model escape from or avoidance of immediate effort. This short-term consequence, however, conflicts with delayed costs: postponed tasks may accumulate, increase later response requirements, reduce opportunities to contact completion-based reinforcement, and contribute to stress accumulation. The game therefore models procrastination as a choice pattern in which immediate relief competes with delayed reinforcement and delayed aversive consequences.

Players personalize the strips by writing a task description (e.g., “answer emails,” “write report,” “workout,” “dinner with friends”).

Each player then places their task strips on the calendar (the board) on the days when they intend to complete them.

Each turn corresponds to one day on the board. Each player receives one default energy unit per day, which can be used to complete all or part of a task. If a player lacks sufficient energy to complete all tasks planned for that day, the incomplete task-days are postponed. If no task is scheduled, that day’s energy unit is lost. Completing an entire task earns one bonus energy unit for later use. After completing all or part of a task, a player draws one card, with a maximum of one draw per day. The card may or may not produce a programmed consequence. The next subsection describes the composition of the card deck.

### 3.1. Card Deck Composition

**Figure 3:**
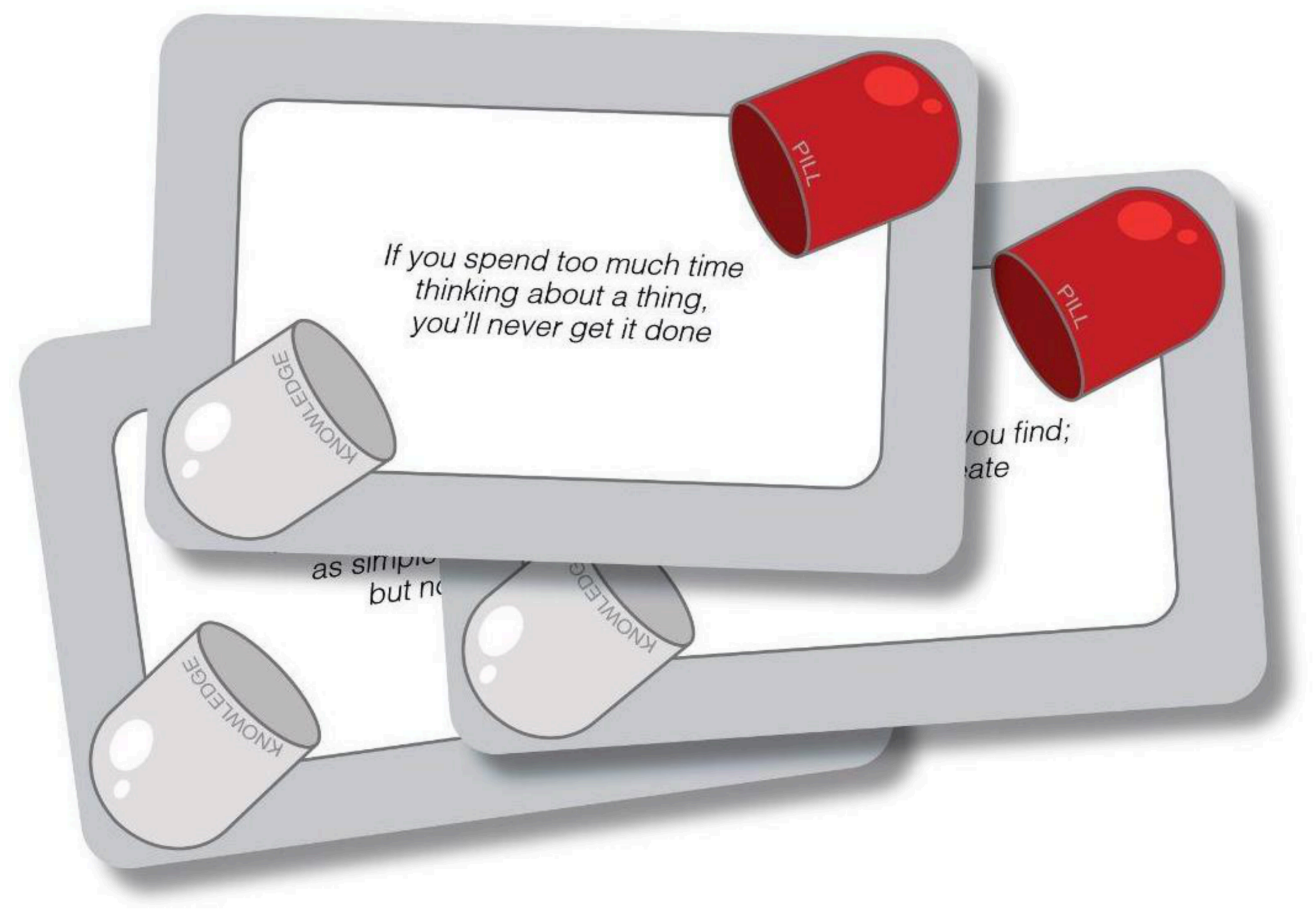
Example of “motivational” card.

The deck includes 56 cards for two players (1 per “day” per player):

Thirty-six motivational cards each present a quotation about time management, productivity, or work–life balance. The author’s name is omitted to reduce possible bias associated with players’ opinions of the author. In landscape orientation, the quotation appears at the center of the card within a cartoon-style illustration of an open medicine capsule. The two halves of the capsule are labeled “knowledge” and “pill” (Figure 3). These cards have no programmed function in the game mechanics and serve as fillers. Exposure to their content is nevertheless intended to prompt reflection on the players’ behavior.

**Figure 4:**
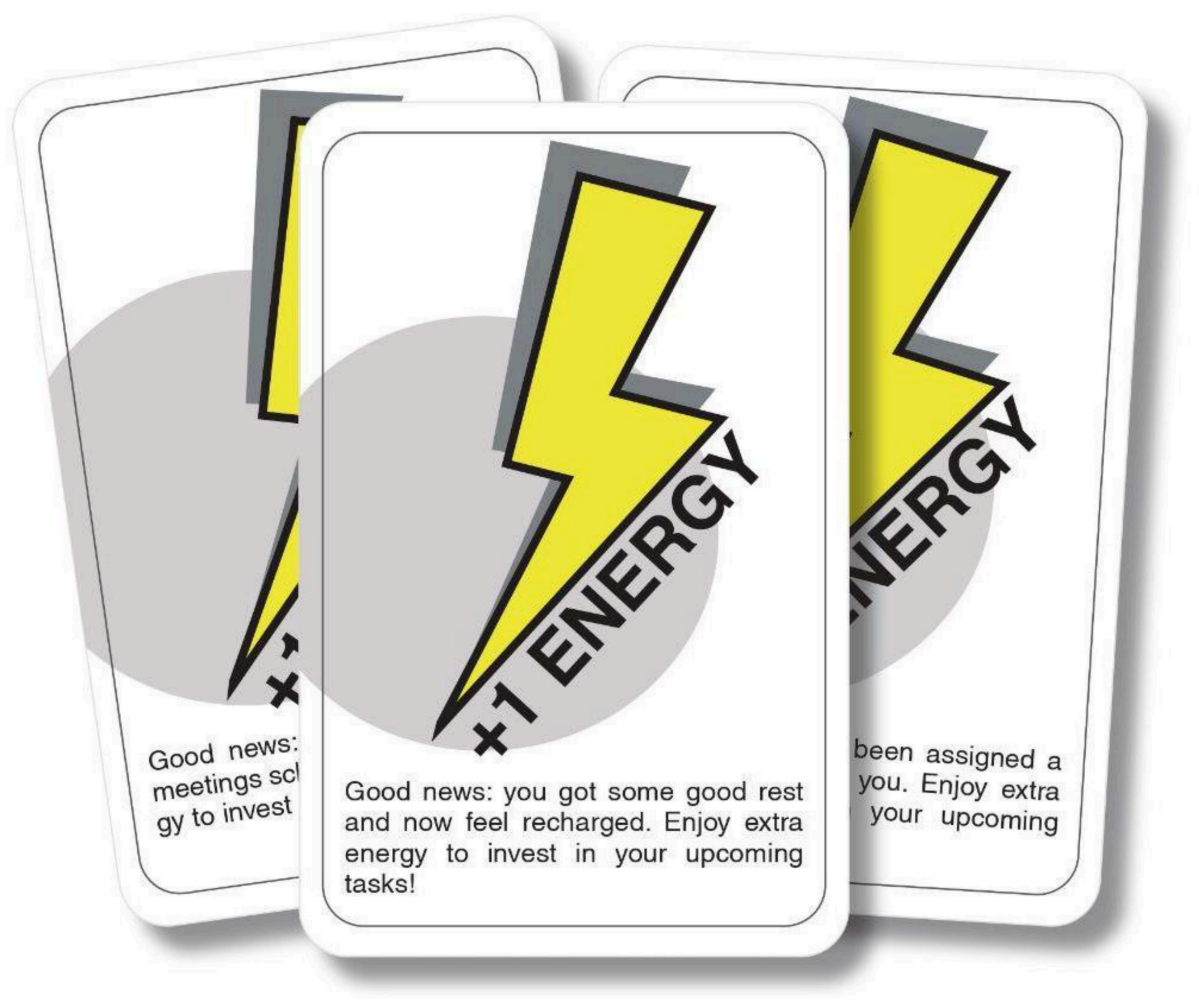
Example of “energy” card.

Twelve energy cards grant one additional energy unit beyond the default daily unit. Each card displays a cartoon-style yellow lightning bolt and the label “+1 ENERGY” (Figure 4). A brief sentence provides a narrative explanation for the additional energy unit but has no other function in the game.

Energy cards deliver units intended to function as positive reinforcers contingent on task execution; players do not draw a card on days when they complete no part of a task. Because the cards are randomly distributed within the shuffled deck, their delivery approximates an intermittent schedule whose exact ratio is determined post hoc. Players also receive an energy unit after completing an entire task and after completing at least one task in each category by the weekly review (see Section 3.2).

**Figure 5:**
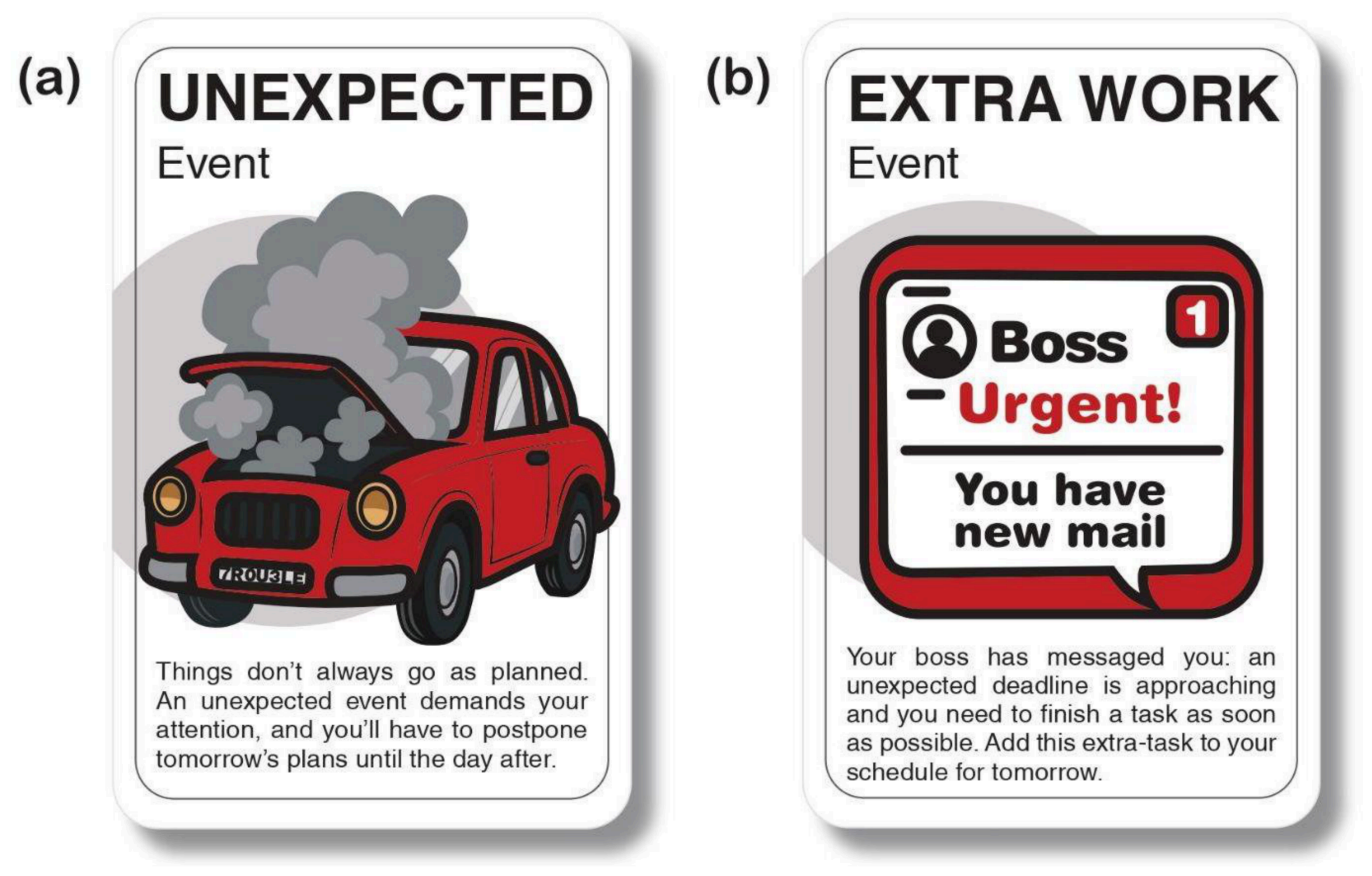

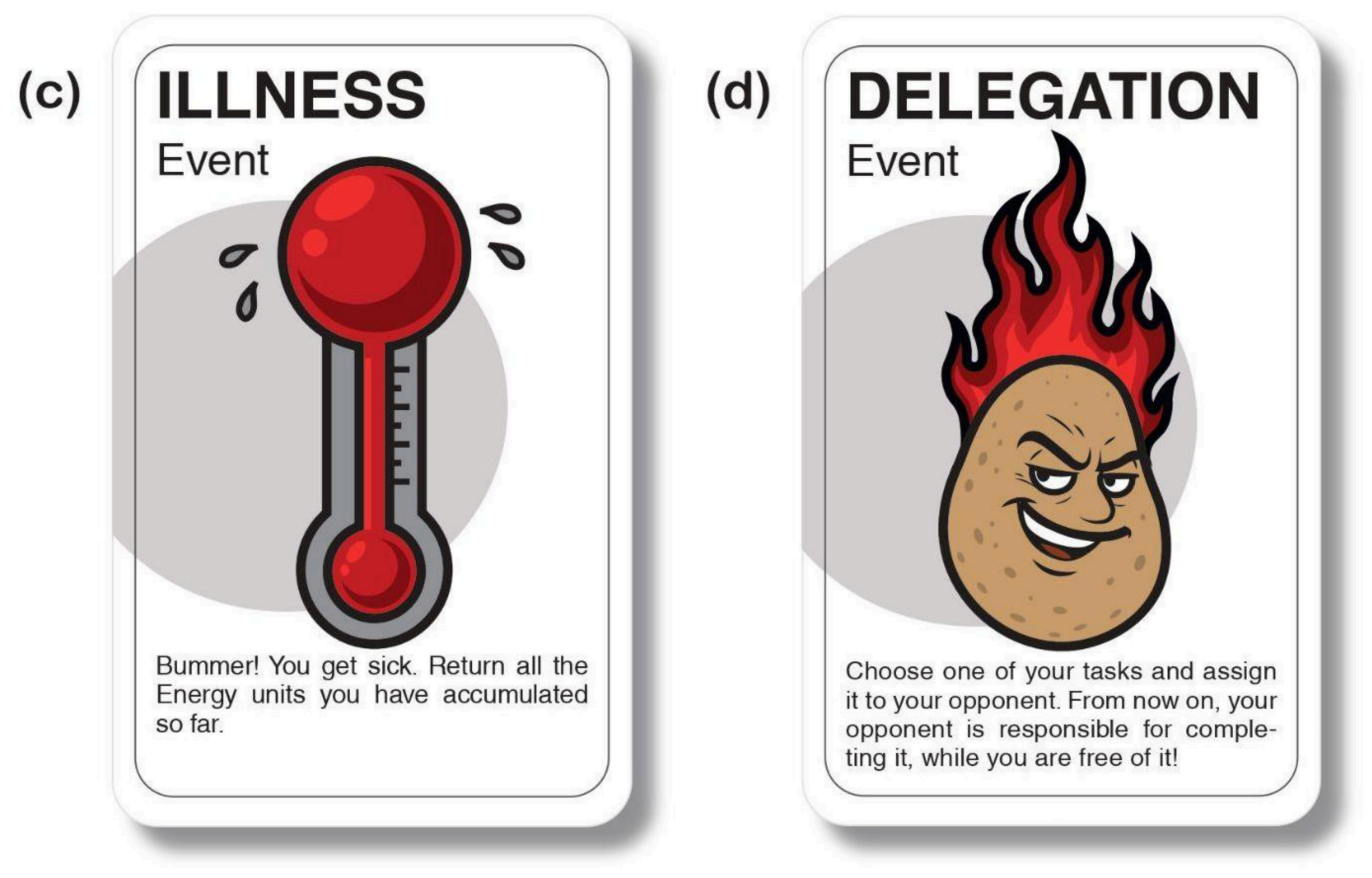
Examples of event cards: Unexpected Event (**a**), Extra Work Event (**b**), Illness (**c**), Delegation Event (**d**).

**Figure 6:**
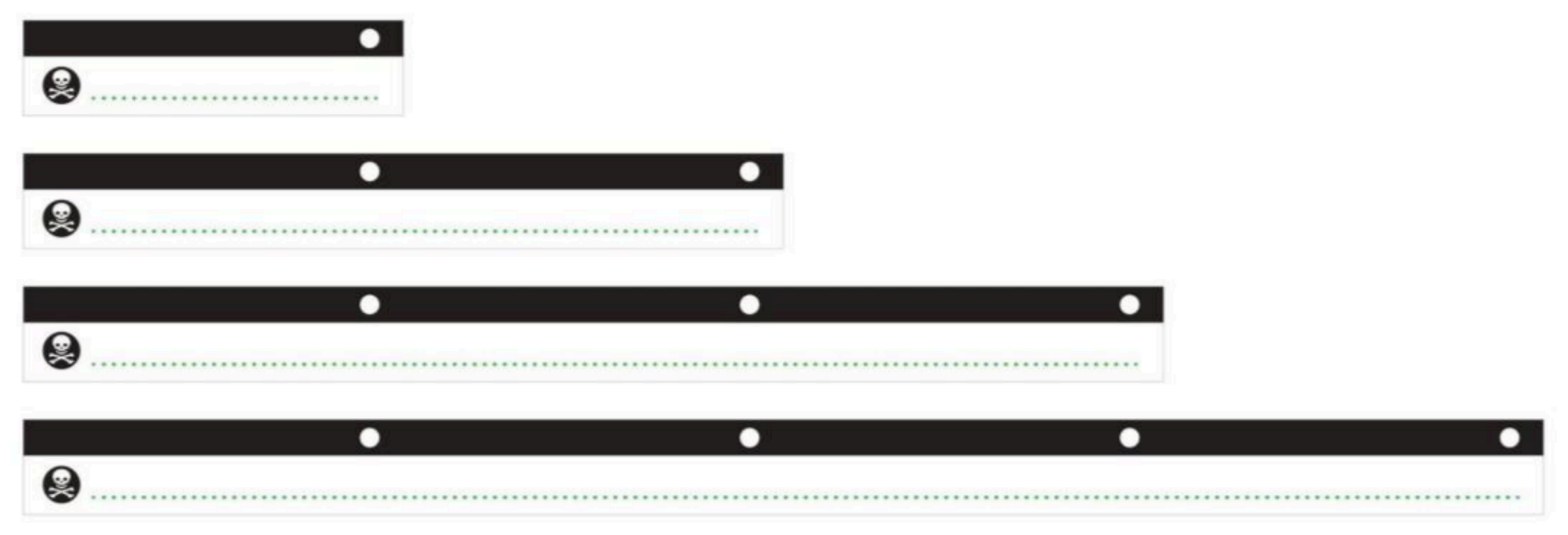
Black version of paper “strips” to be used when extra work and delegation events occur.

The deck also contains “event” cards:

1. Two cards that add an unplanned 1-day delay due to an unexpected event, forcing a schedule shift. These cards display the text “Unexpected Event”, accompanied by a cartoon-style illustration of a car that has broken down (Figure 5a) and the following description: “Things don’t always go as planned. An unexpected event demands your attention, and you’ll have to postpone tomorrow’s plans until the day after”.
2. Two cards that add an extra one-day work task due to a last-minute deadline. These cards display the text “Extra Work Event”, accompanied by a cartoon-style illustration of an urgent email notification from the boss (Figure 5b) and the following description: “Your boss has messaged you: an unexpected deadline is approaching and you need to finish a task as soon as possible. Add this extra-task to your schedule for tomorrow”. Extra work tasks are represented by black strips (Figure 6).
3. Two Illness cards cause the loss of all accumulated energy units. Each card displays the text “Illness Event,” accompanied by a cartoon-style illustration of a thermometer at the top of the scale (Figure 5c), and the following description: “Bummer! You get sick. Return all the Energy units you have accumulated so far”.
4. Two wild cards allow a player to transfer one of their own tasks to the opponent, simulating delegation or the “buck passing” sometimes observed in organizations. These cards display the text “Delegation Event,” accompanied by a cartoon-style illustration of a hot potato and the following description: “Choose one of your tasks and assign it to your opponent. From now on, your opponent is responsible for completing it, while you are free of it!” (Figure 5d). This arrangement may function as negative reinforcement for the delegator if removal of the task increases the future probability of delegation; for the recipient, it introduces additional aversive task demands. Delegated tasks are represented by black strips (Figure 6).

The deck includes 56 cards for two players (1 per day per player): 8 event cards, 12 energy cards, 36 motivational/tip cards. The resulting reinforcement schedule is determined post hoc by the order of cards after shuffling.

### 3.2. Weekly Review

At the end of each week, players land on a weekly review space. If a player has completed at least one task in each category, they receive a bonus energy unit. If work tasks spill into the weekend, a stress unit is also assigned.

During the review, players privately answer a brief questionnaire:

- What would you change in your planning?
- What worries you about next week?
- How effective did you feel this week? (scale 1–5)
- How effective do you expect to be next week? (scale 1–5)

The weekly review is described here because it is a core game mechanic, not merely a pilot-study procedure. It arranges self-monitoring prompts and feedback opportunities, and it supports rule-governed strategy adjustment across repeated sessions.

At the end of the game, both players discuss their reflections and strategies.

The first stress unit does not increase task cost. Beginning with the second stress unit, task costs are multiplied by 2, 3, 4, and so forth. Players may remove stress units by spending one energy unit per stress unit, but only at the end of a turn, thereby creating a strategic trade-off.

The game ends after 32 turns. The final score was calculated by adding one point for each task-day completed, one point for each unused energy unit remaining after the final turn, and subtracting one point for each stress unit.

In summary, the reinforcement system within Steady State operates across three concurrent schedules. First, a variable-ratio-like schedule governs the delivery of energy cards, reinforcing partial task completions with intermittently distributed, yet behavior-contingent, consequences. Second, energy units are delivered contingent on full task completion. Within each task, energy delivery follows a fixed completion requirement, although this requirement varies across tasks from one to four task-days. Finally, at the macro level, a differential reinforcement system promotes balanced engagement across life domains (e.g., work, health, relationships), reinforcing patterns of behavior that achieve predefined distributions. This compound system reflects naturally occurring contingencies of reinforcement that shape and maintain complex time management repertoires.

Table 2 shows how the characteristics identified in Morford et al.’s (2014) functional account of game-playing map onto the features of Steady State.

## 4. Pilot Study

This pilot phase was designed as a proof-of-concept demonstration. Its purpose was to examine whether Steady State generates measurable and behaviorally interpretable in-game patterns under contingent versus largely noncontingent arrangements. It was not designed to establish educational effectiveness, generalization, or transfer to everyday time-management behavior. These aspects require a separate empirical evaluation informed by the present pilot.

Because the dependent variables exist only within gameplay, they cannot be measured during a no-game baseline. We therefore used an alternating treatments design (ATD), which permits rapid, session-by-session comparisons across short sessions without an extended baseline phase (Manolov & Onghena, 2018).

The two alternating conditions were the full version of Steady State (Condition A) and a procedural comparison version in which the main response–consequence relations were minimized and energy availability and stress accumulation were largely determined by stochastic outcomes (Condition B).

The present pilot study examined only one dyad. This choice was appropriate for the present paper because the primary aim was technological description and proof-of-concept evaluation of the game mechanics, not experimental demonstration of a replicated effect. Replication with multiple dyads, formal single-case analyses, and counterbalanced or rotated condition sequences will be required in the full evaluation. The study protocol was approved by the Ethics Committee for Transdisciplinary Research, Sapienza University of Rome [CERT_19BDB31B323].

The expected results of this pilot study were more differentiated patterns of planning, allocation, postponement, and stress management under the contingency-based version of Steady State than under the stochastic procedural comparison condition. Specifically, Condition A was expected to produce more strategy-dependent patterns than Condition B, as reflected in planning time, task postponements, end-of-session unused energy, stress accumulation, and task-distribution balance across sessions.

## 5. Methods

### 5.1. Participants

Two university students (one woman and one man, both 25 years old) volunteered for this pilot study. Participants were recruited through a course announcement at Sapienza University of Rome. Before participation, they were interviewed about self-reported time-management difficulties.

During the pre-study interview, Player 1 reported difficulties related to perfectionism, high standards, delayed task initiation, and feeling overwhelmed when managing multiple commitments. Player 2 reported fewer procrastination difficulties, a tendency to start tasks promptly, and strong work involvement, although with a possible imbalance toward work-related activities. These self-reported differences were used only to describe the pilot sample and to contextualize exploratory observations; they were not treated as diagnostic categories or independent variables. From a behavioral perspective, such differences may reflect distinct learning histories related to task completion, deadline management, and the consequences associated with work-related behavior.

Both participants provided written informed consent prior to participation.

### 5.2. Materials

Condition B used the same board and default daily energy unit as Condition A, but its cards had gray backs so that the experimental condition could be identified immediately. Except for the differences listed below, the two conditions used the same core rules. Particularly, players in condition B:

- Do not obtain energy units upon completion of tasks;
- Do not receive the weekly energy bonus for balancing task categories;
- Do not draw event or energy cards from the deck, which contains only motivational cards in this condition;
- Receive a weekly energy reserve every Monday based on a roll of two dice. The reserve equals the sum rolled and is delivered independently of the player’s behavior. Any energy remaining at the end of the week is returned;
- Flip a coin during each weekly review to determine whether they receive one stress unit.

Thus, in Condition B, the main consequences relevant to energy availability and stress accumulation were largely determined by chance rather than by the player’s planning or task-execution behavior.

Condition B was designed as a procedural comparison version rather than as an entirely different game. The board, task strips, temporal structure, and core response requirements, including planning, task allocation, execution, and postponement, were intentionally retained across conditions. Requiring participants to learn two substantially different rule systems would have introduced additional differences in rule-learning demands, procedural fluency, and cognitive load, thereby making the conditions less directly comparable. Maintaining a common procedural framework also preserved the possibility that planning and allocation repertoires could generalize across closely related game contexts, which is consistent with the educational rationale of the game. At the same time, this procedural overlap created the possibility of cross-condition carryover, which is considered in the interpretation of the pilot findings.

The card deck used in Condition B includes only motivational quotes. Therefore, the predetermined sequence of condition alternation was independent of the within-game distribution of adverse events. In Condition A, players drew cards from a common deck that was shuffled before each game. The deck contained 8 adverse-event cards out of 56 cards (14.3%), and each player had up to 28 card-drawing opportunities during a full game. The deck was reset and shuffled before each game. Cards were not replaced during a game. Because players drew a card only after completing all or part of a task, the deck was not necessarily exhausted in every session; therefore, not all eight adverse-event cards necessarily appeared. Accordingly, before the start of each game, both players had the same expected exposure to adverse events, although random drawing could result in an unequal distribution between players within an individual session. To maintain the same deck size, 20 additional quotations were added to those used in Condition A. These quotations also omitted the authors’ names to reduce possible bias associated with participants’ opinions of the authors.

Condition B was visually distinguishable from Condition A by design (Barlow & Hayes, 1979): the card decks differed in color, and two energy dice and a stress coin were available only in Condition B. In an alternating treatment design, the conditions should be readily discriminable.

### 5.3. Procedure

The participants received oral and written instructions for both versions of the game. Over three weeks in December 2025, the dyad completed 12 sessions, alternating between Conditions A and B according to the fixed sequence BAABABBABABA.

The sequence was selected a priori as a pragmatic constrained sequence rather than a randomized or fully counterbalanced schedule. It provided six exposures to each condition, with three exposures to each condition in each half of the study, and limited runs of the same condition to no more than two consecutive sessions. The first-order transitions were BA = 5, AB = 4, AA = 1, and BB = 1. Thus, the sequence permitted frequent comparisons but was only approximately balanced for immediate condition history and was not intended to eliminate carryover.

The procedural similarity of the conditions was intentional and distinct from the choice of sequence. The common game structure minimized differences in rule learning, procedural familiarity, and task demands. Nevertheless, because the same core responses were available in both versions, temporary cross-condition generalization of response patterns, rules, or expectations could not be excluded. The initial B session provided one observation before exposure to Condition A, but a single observation cannot establish a stable baseline. No washout period was included, and no return to baseline was assumed. Comparisons were therefore interpreted as descriptive within-participant contrasts under alternating exposure, not as estimates of isolated treatment effects.

After the last session, participants took part in a brief, unstructured debriefing aimed at gathering general feedback about their experience with the activity. The exchange was not recorded, transcribed, coded, or treated as a formal qualitative data source.

### 5.4. Dependent Variables and Measurement

All dependent variables were measured within each session using video recordings obtained from a bird’s-eye view of the board and session artifacts, including the calendar board and task strips.

1. Planning duration (seconds). Defined as the elapsed time from the moment the player began planning the session (first contact with task strips or board for planning purposes) to the first executed move of Turn 1. Measured from video timestamps.
2. Total session duration (seconds). Defined as the elapsed time from the beginning of the planning phase to the completion of the final turn. Session duration was derived from video timestamps and was examined descriptively as an indicator of procedural differences between the two game versions, rather than as a measure of educational effectiveness or strategy quality.
3. Delays (cumulative count). This variable was defined as the cumulative number of one-day postponements applied to planned task-days, regardless of whether the postponement resulted from the player’s allocation choices or was directly imposed by an event card. It is not the number of distinct postponed tasks. For example, if one task-day was moved forward by three days, three task-day shifts were counted. Thus, values can exceed the number of turns because they represent cumulative postponement units across all task-days in a session. Accordingly, this measure represents the total in-game postponement burden and should not be interpreted as a pure measure of planning or time-management skill.
4. Unused energy (count). Energy left at the end of each session.
5. Stress units (count). Defined as the number of stress units accumulated and remaining at the end of the session.
6. Task distribution balance (rate). Defined as the proportion of weekly reviews at which the player had completed at least one task in each category (Work, Health/Wellbeing, and Relationships/Leisure).

### 5.5. Data Extraction and Verification

One author extracted all values from the session videos and artifacts. The second author verified those values against the video timestamps and final board states, and any discrepancies were resolved through joint review. Because the measures involved explicit game-state outcomes and discrete observable events, the coding required little subjective judgment. However, the verification procedure was not a fully independent coding protocol, so a formal interobserver agreement statistic could not be calculated retrospectively.

### 5.6. Descriptive Analysis

Given the exploratory nature of this pilot study, session-level changes were examined descriptively. A potential strategy change was considered only when a departure from the player’s previously observed allocation pattern occurred across more than one decision opportunity or recurred in a subsequent session. These changes could involve task prioritization, energy expenditure, or resource conservation. Isolated choices were described as behavioral novelties rather than as evidence of a stable strategy change. Because these criteria were applied retrospectively and no independent coding was conducted, the interpretations should be regarded as tentative.

## 6. Results

Given the demonstrative, single-dyad purpose of the pilot and the small number of sessions per condition, the results are presented descriptively and through structured visual inspection. Formal randomization tests or non-overlap indices were not used to support inferential claims because the pilot was not designed to establish a replicated treatment effect.

For clarity, a session refers to one complete game, a turn to one day on the board, and a week to seven consecutive turns. For each player and session, we plotted the energy units retained at the end of each turn and the cumulative number of task shifts in both conditions. The session-level unused-energy measure was the average of energy units remaining after the final turn.

Visual inspection suggested that, in Condition A, Player 1 and Player 2 showed more differentiated performance patterns, whereas in Condition B their curves were more similar and more strongly constrained by stochastic resource availability.

Both players required progressively less time to plan across sessions. Mean planning durations in Conditions A and B were 262 and 190 s, respectively, for Player 1 and 271 and 153 s for Player 2. From the first to the last session in Condition A, planning duration decreased by 50 s for Player 1 and 45 s for Player 2. In Condition B, it decreased by 35 and 60 s, respectively. Planning was consistently faster in Condition B, as expected because that condition reduced the functional value of optimizing task arrangements to avoid programmed consequences.

Total session duration also differed descriptively between conditions. Condition A sessions lasted, on average, 1809 s (SD = 474; range = 1380–2659), whereas Condition B sessions lasted, on average, 1008 s (SD = 219; range = 670–1240). Thus, sessions in Condition A were consistently longer than those in Condition B. Given the demonstrative nature of the pilot and the small number of sessions, this difference was not subjected to inferential analysis.

Table 3 shows the players’ task-category balance rates in Condition A. With few exceptions, both players completed at least one task in each category every week.

**Figure 7:**
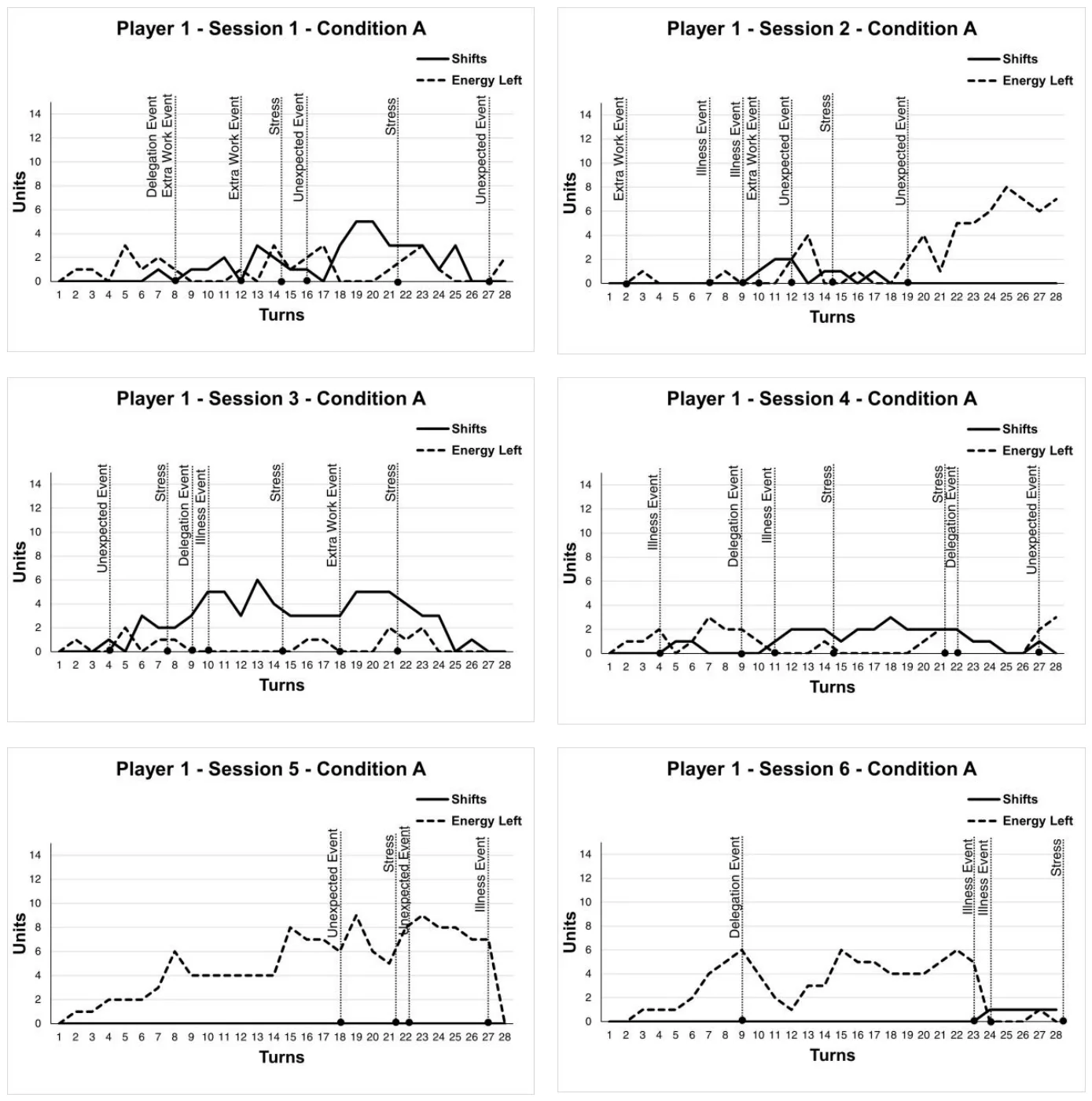
Player 1’s plots for Condition A.

**Figure 8:**
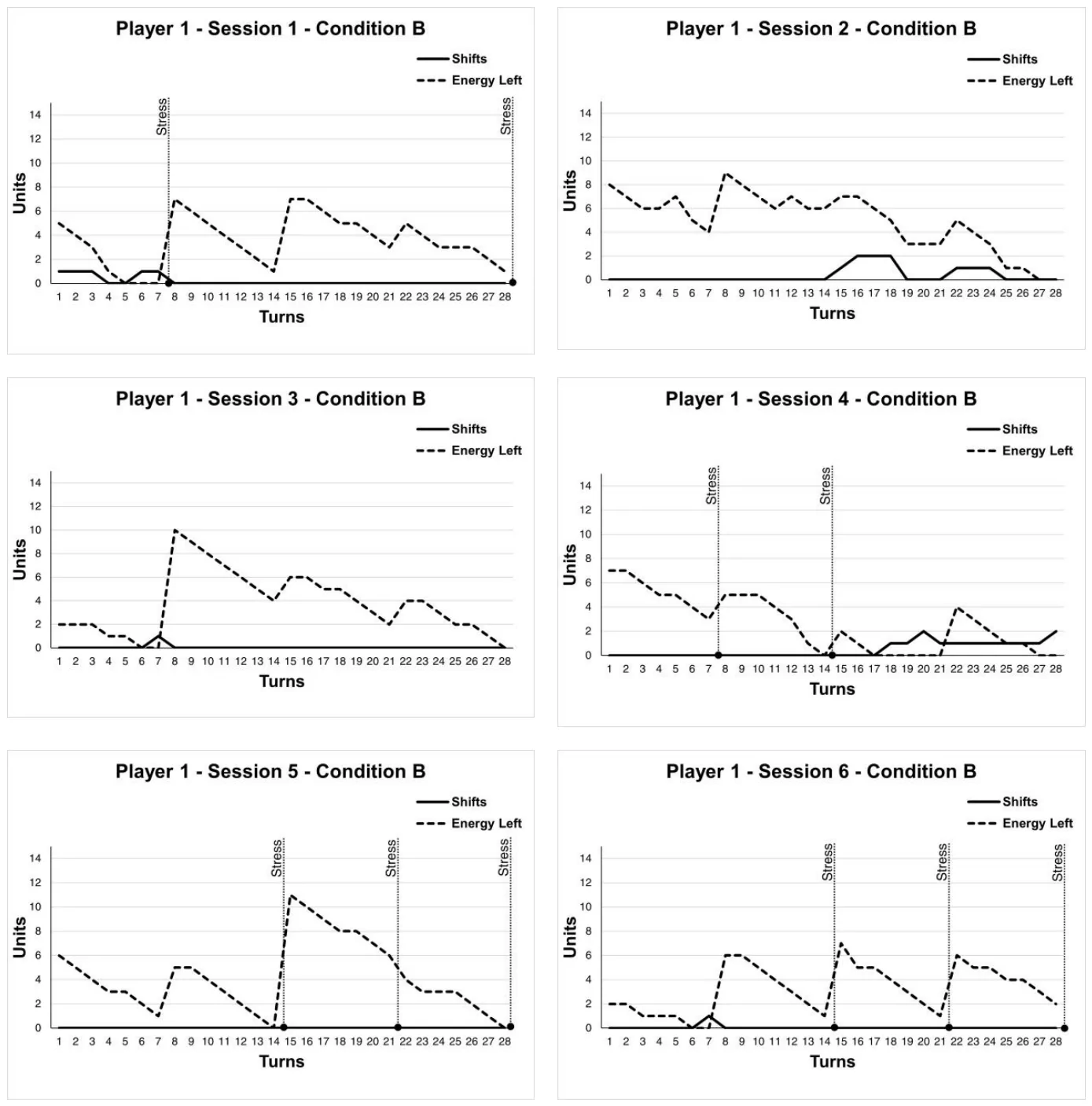
Player 1’s plots for Condition B.

**Figure 9:**
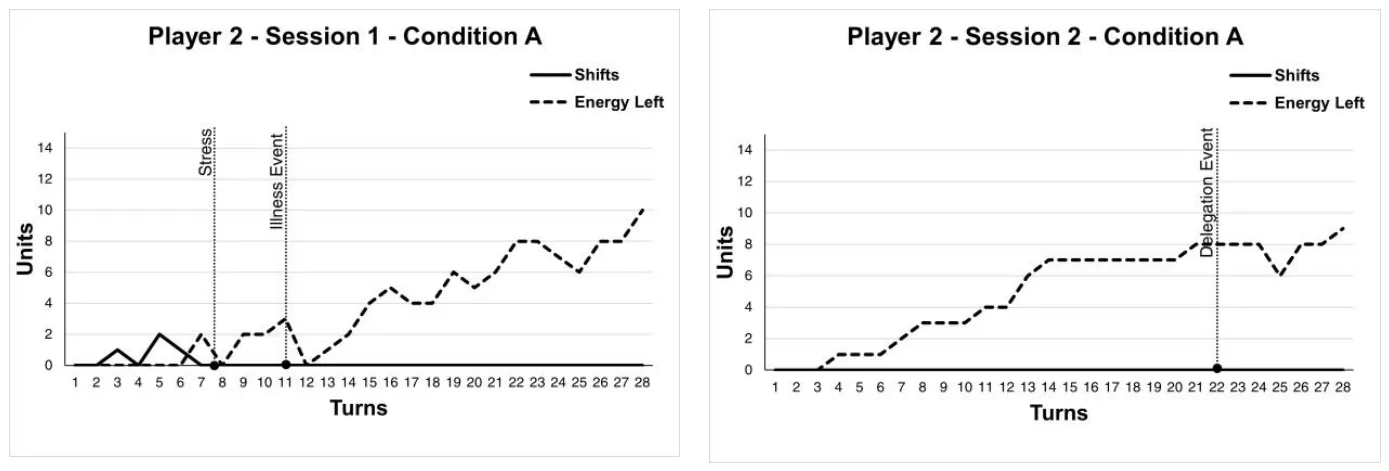

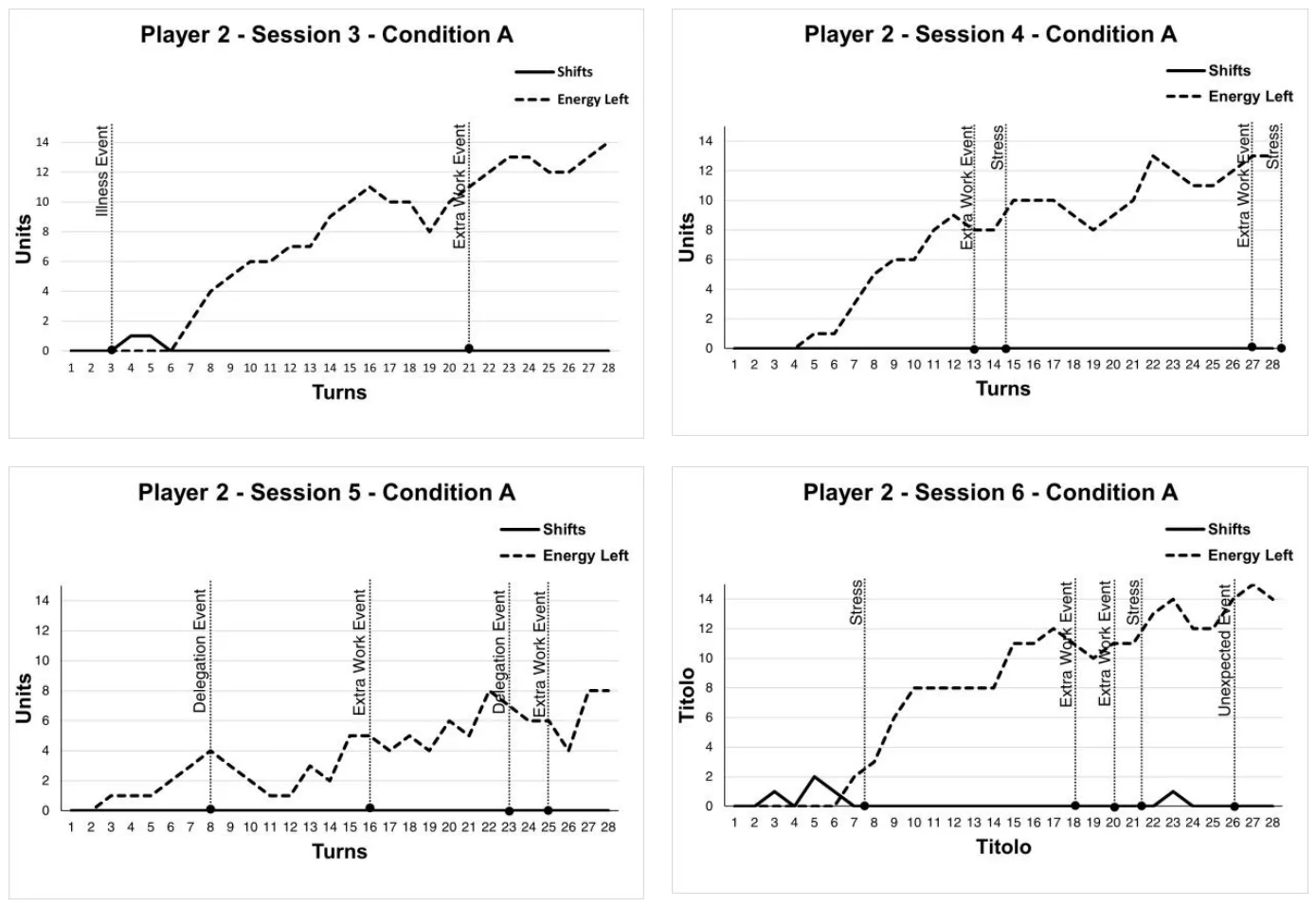
Player 2’s plots for Condition A.

**Figure 10:**
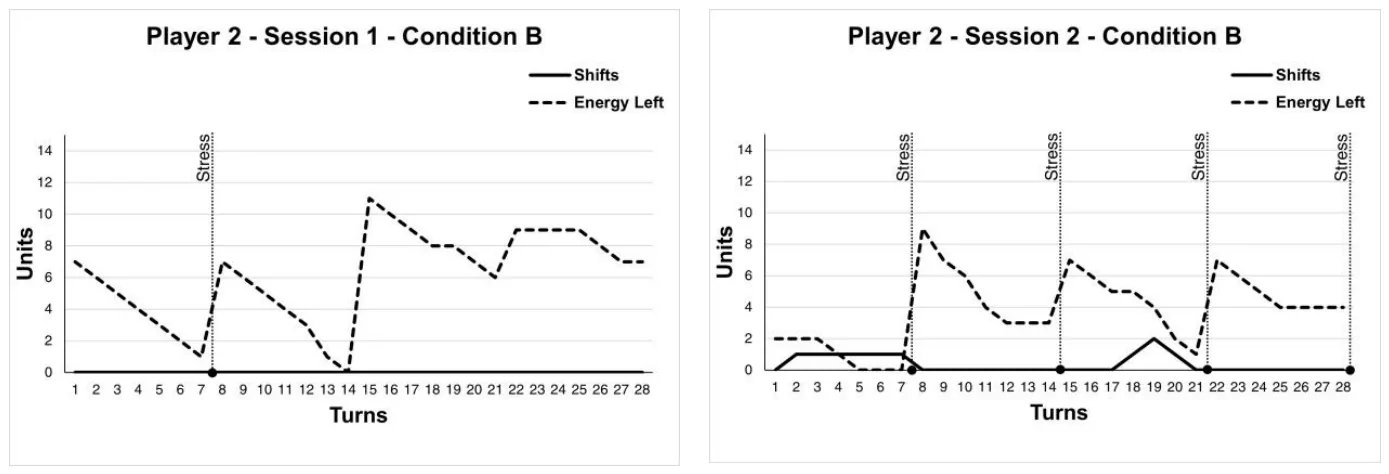

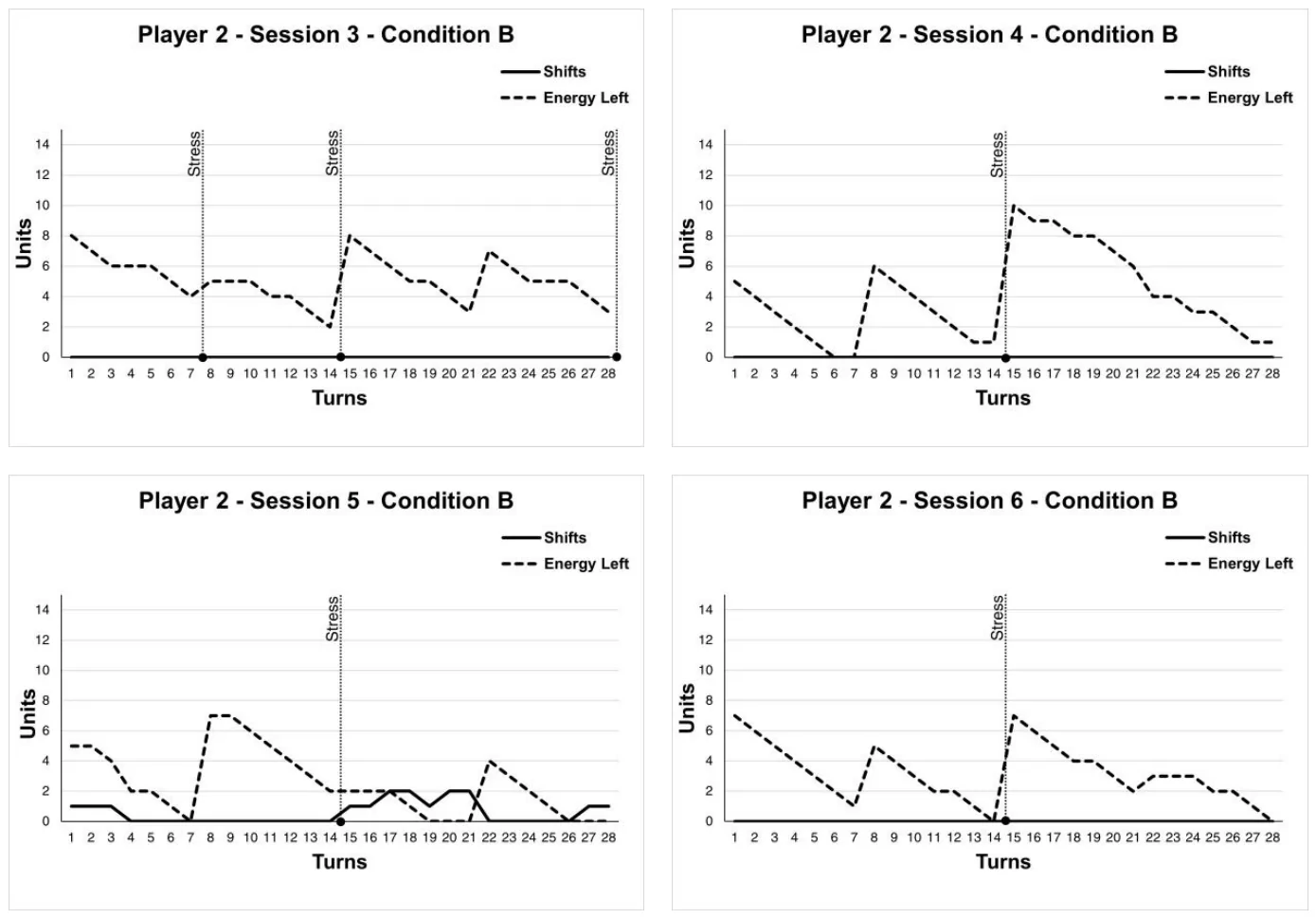
Player 2’s plots for Condition B.

Figure 7, Figure 8, Figure 9 and Figure 10 present, for each player and condition, cumulative task shifts, indicating postponement, and the energy reserve retained at the end of each turn. The energy curves show the trajectory of resource availability across the session. By contrast, the unused-energy values correspond only to the energy remaining after the final turn.

### 6.1. Player 1’s Performance in Condition A

In the early sessions, Player 1 showed an unstable pattern characterized by repeated postponements, energy remaining available despite executable tasks, and difficulty responding to disruptions. In Sessions 1 and 2, several aversive events occurred, including delegation, illness, unexpected events, and extra work. These events reduced the player’s margin for error and contributed to increases in task shifts and stress accumulation. However, the observed pattern was not attributable to chance alone: at several points, energy was left unused despite executable tasks being available, and non-work tasks were repeatedly postponed in favor of preserving resources for work-related demands. Taken together, the presence of available energy, executable tasks, and repeated postponements suggests an initially conservative allocation pattern that was not consistently adaptive under the game’s contingencies.

Across the middle sessions, Player 1 made choices consistent with a possible change in allocation strategy. In Session 3, despite high cumulative shifts and stress, the player departed from the earlier work-prioritization pattern by completing a short nonwork task before longer work tasks and thereby contacted the immediate consequence associated with task completion. This single episode, however, does not establish a strategy change. In Session 4, energy expenditure became more consistent, and resources were allocated more flexibly across task types, although the player still tended to conserve energy when nonwork tasks were scheduled.

In Sessions 5 and 6, resource allocation was relatively more stable. Shifts remained at zero for most of Session 5, and adverse events produced fewer observable disruptions in task completion. In Session 6, some conservative choices appeared functional within the event sequence, such as retaining energy during one turn and using it to complete additional tasks on the next turn.

Taken together, these patterns may indicate increasing sensitivity to the game’s contingencies. However, the single-dyad design, retrospective criteria, and lack of independent assessment prevent these changes from being distinguished clearly from session-to-session fluctuation. Performance also remained sensitive to the timing and density of adverse events.

Table 4 reports the number and type of adverse-event cards drawn by Player 1 across Condition A sessions. The distribution of events varied across sessions, with a higher number of aversive events occurring in the early sessions. These data are provided to document the session-by-session exposure to stochastic perturbations and to support interpretation of the descriptive performance patterns.

Figure 7 shows Player 1’s performance in Condition A.

### 6.2. Player 1’s Performance in Condition B

In Condition B, Player 1’s performance provided less information about strategy because energy availability and stress accumulation were largely noncontingent. Fluctuations in energy and task shifts mainly followed dice and coin outcomes rather than task-completion behavior. The plots therefore show how performance varied when the functional relation among planning, task execution, and consequences was minimized. Unlike in Condition A, individual strategy showed little evidence of producing stable or cumulative changes across sessions.

Figure 8 shows Player 1’s performance in Condition B. In this condition, energy availability was determined primarily by dice rolls, and stress accumulation by coin tosses during weekly reviews.

Because category balance was not programmed as a reinforcing criterion in Condition B, no corresponding balance table is reported for this condition.

### 6.3. Player 2’s Performance in Condition A

Player 2 showed comparatively low postponement and rapid recovery from disruptions from the outset. In Session 1, a few early shifts appeared, likely reflecting initial adjustment to the rules and planning demands, but these were rapidly resolved. Even when an Illness Event eliminated accumulated energy, the player recovered without sustained increases in postponement. In Session 2, the pattern remained stable: shifts were minimal or absent. Accumulated energy increased progressively, and greater resource availability preceded later recovery from disruptions.

Across the middle sessions, Player 2’s performance remained robust to perturbation. In Session 3, an early Illness Event temporarily reduced available resources, but the player quickly recovered and returned to a stable pattern of task completion and energy accumulation. Extra Work Events, which could have functioned as aversive disruptions, were generally managed without substantial delay because sufficient energy had already been accumulated. In Session 4, a similar pattern appeared: stress units generated by weekend work spillover were rapidly removed and did not produce sustained disruption. This pattern is consistent with a possible buffering function of accumulated energy: energy available before adverse events was followed by rapid recovery and limited task postponement. This interpretation is based on the temporal relation among resource availability, adverse events, and subsequent performance, rather than on the unused energy count alone.

In the later sessions, Player 2 continued to show a stable and adaptive pattern. In Session 5, despite exposure to multiple aversive events, shifts remained near zero, suggesting that the player’s performance was not simply due to favorable event distribution. In Session 6, some early delays occurred under low energy availability, but the player again recovered and ended with a high level of stored energy. Overall, Player 2’s trajectory was characterized by rapid recovery from disruptions, low postponement, and progressive accumulation of energy, consistent with sustained contact with the game’s programmed contingencies.

Table 5 reports the number and type of aversive-event cards experienced by Player 2 across Condition A sessions. As for Player 1, exposure to aversive events varied across sessions. The table is included to document the distribution of stochastic events during Condition A and to provide contextual information for the descriptive analyses.

Figure 9 shows Player 2’s performance in Condition A.

### 6.4. Player 2’s Performance in Condition B

In Condition B, Player 2 showed fluctuations that were primarily linked to stochastic resource availability rather than to stable differences in planning strategy. As for Player 1, energy reserves were determined by dice rolls, while stress accumulation was determined by coin tosses during weekly reviews. Consequently, this condition reduced the opportunity for player-specific strategies to contact programmed consequences. The resulting curves provide a useful comparison with Condition A. In Condition A, Player 2 showed stable recovery, resource accumulation, and little postponement; in Condition B, performance was more externally constrained by chance outcomes. 

Figure 10 presents the corresponding plots for Player 2 in Condition B.

The results for the main dependent variables are summarized separately for each player in Table 6 (Player 1) and Table 7 (Player 2).

## 7. Discussion

The findings are discussed in relation to the two objectives and the exploratory research questions. O1 was addressed through the technological description and functional mapping of the game mechanics presented in Section 2 and Section 3. The pilot findings address O2 and the associated exploratory research questions.

This pilot evaluated whether Steady State’s contingency-based gameplay (Condition A) produced more behaviorally interpretable in-game patterns than a procedural comparison condition in which the main response-consequence relations were minimized and stochastic outcomes were emphasized (Condition B). The most conservative interpretation is that the game design can generate behaviorally meaningful variability in planning and allocation measures under contingent versus largely noncontingent arrangements. Although the present data are limited to a single dyad and are primarily demonstrative, the observed patterns are broadly consistent with the functional assumptions guiding the game design, while also highlighting the non-trivial influence of stochastic events.

With respect to RQ1, the embedded measures captured session-by-session variability in planning duration, task postponements, energy allocation, stress accumulation, and total session duration. However, their interpretation was affected by stochastic events and by the procedural differences between conditions.

Condition B served as a useful procedural comparison because fluctuations were driven largely by stochastic energy and stress outcomes, and between-player differences were attenuated. This pattern suggests that the embedded measures, such as task shifts, unused energy, and stress accumulation, are sensitive to whether consequences are related to player behavior or primarily determined by chance. When the functional relation between planning or execution and outcomes is minimized, individual strategies have fewer opportunities to produce stable performance differences.

Condition B should not, however, be interpreted as a control that isolates reinforcement or response-consequence coupling. Although the conditions shared the board, task materials, temporal structure, and core responses, they differed in rule complexity, the number and functional relevance of decisions, the effective strategy space, and session duration. Condition A sessions were descriptively longer, consistent with their additional decisions, programmed consequences, event-management requirements, and opportunities for strategic adjustment. The contrasts between conditions therefore cannot be attributed uniquely to reinforcement processes.

The procedural overlap also has different implications for educational design and experimental control. Generalization of planning and allocation repertoires across closely related contexts may be educationally desirable, but it may reduce the functional independence of successive condition comparisons. Because both versions used the same board, task strips, temporal structure, and core responses, patterns or rules developed in Condition A may have generalized temporarily to subsequent B sessions. Such generalization would be most relevant to behaviors available in both versions, including planning duration, task ordering, and postponement, and could reduce visual separation between conditions.

Carryover in the opposite direction is also possible. Exposure to stochastic outcomes in Condition B may have temporarily reduced the perceived utility of strategic responding in a subsequent A session. Alternatively, the clear contrast between conditions may have increased sensitivity to the contingencies upon returning to Condition A. The present single-sequence pilot cannot distinguish among these possibilities.

By contrast, Condition A produced patterns consistent with possible adaptation to the contingencies, although these interpretations remain exploratory given the single-dyad design. Planning time decreased across sessions in both conditions, possibly reflecting procedural familiarity and the development of game-specific strategies and heuristics across repeated play (Morford et al., 2014). Planning time was consistently shorter in Condition B, which is expected because optimization has limited utility when reinforcement and aversive stimulation are largely noncontingent.

Programmed consequences in Steady State were arranged through a compound system: energy cards were delivered intermittently following partial task progress, while full task completions consistently produced energy units. The game also arranged a weekly bonus contingent on balanced engagement across life domains, creating a differential consequence for allocation patterns rather than only for raw productivity (Ferster & Skinner, 1957; Skinner, 1953). This combination was intended to approximate the layered contingencies that sustain effective time management in everyday settings (immediate but variable payoffs for progress, reliable payoffs for completion, and broader incentives for balanced responding). It also provides a plausible functional account of why performance in Condition A could reflect strategy and learning more strongly than in Condition B, the stochastic procedural comparison condition.

A central point for interpreting Condition A is the role of chance as a perturbation layered on top of skill. The deck includes event cards that can abruptly alter resource availability, task demands, schedule requirements, and response costs and, in some cases, may also modify motivating operations (Michael, 1993). These events are intentionally stochastic to model real-world productivity contexts where disruptions occur unpredictably. However, in a small-n pilot, unequal exposure to adverse events can materially affect session outcomes, especially when events cluster at sensitive points of the “workweek” sequence.

This is particularly evident in Player 1. In early sessions, Player 1 experienced a high rate of aversive events (as noted in Table 4 in the Results: for example, five adverse-event cards in Session 1 and six in Session 2). In such circumstances, poorer outcomes are plausibly overdetermined: they reflect both (a) less efficient allocation strategies (for example unused energy in periods where executable tasks were available, repeated postponement of non-work tasks to “save” energy) and (b) unfavorable event exposure that reduces the margin for error and amplifies the cost of suboptimal choices. The key implication is that Player 1’s early performance should not be interpreted as a low-skill pattern alone: it is better conceptualized as performance under contingencies with unusually frequent disruptions that created a harsher learning environment.

At the same time, the data suggest that stochastic adversity did not fully account for Player 1’s trajectory. Across later sessions, Player 1 showed patterns more consistent with strategy change and improved adaptation. The emergence of a new strategy (prioritizing a short non-work task to secure completion-based energy) is consistent with shaping by immediate outcomes and with the game’s design goal of making the benefits of “small wins” salient. Improvement in Sessions 5 and 6 suggests that once the player began contacting a more stable reinforcement pattern (and possibly experienced a less punishing run of events), performance became more robust, including better handling of disruptions when they did occur.

Player 2 displayed a comparatively stable and adaptive pattern in Condition A, often characterized by rapid recovery from disruptions and near-zero postponements across most sessions. This pattern is consistent with a possible cycle in which task completion was followed by greater resource availability, and available resources were in turn associated with more rapid recovery from later disruptions. The descriptive evidence for this interpretation comes from the sequence of energy accumulation, adverse events, and subsequent responding, not from a high end-of-session energy value by itself. From a behavior-analytic standpoint, this pattern is compatible with the possibility that contingent energy gains reinforced task engagement and completion. However, because changes in the future probability of these responses were not isolated, the reinforcing function of energy gains was not directly demonstrated in the present study (Skinner, 1953). Testing this interpretation would require sequential analyses of energy delivery, subsequent task engagement, and responses to adverse events.

With the exception of the delegation card, Steady State intentionally minimizes inter-player contingencies. Avoiding interdependent or competitive contingencies keeps the functional focus on self-management and rule-governed reflection rather than on social competition. Programmed randomness through event and energy draws serves a dual purpose: it models intermittent reinforcement and unsignaled shifts in establishing operations that characterize natural productivity contexts, and it creates perturbations that test resilience of allocation strategies. In the present pilot, this also implies a methodological consideration: unequal exposure to adverse events can meaningfully affect short-run outcomes (as suggested by Player 1’s early sessions), so future studies should consider procedures that balance or yoke event exposure across players or sessions. The delegation card preserves a minimal and ecologically plausible social perturbation that occurs in real settings. For the delegator, task transfer removes an immediate task demand and would function as negative reinforcement if that removal increased the future probability of delegation. For the recipient, it introduces an additional task demand. This mechanic adds ecological plausibility, but its social validity was not formally assessed (Michael, 1975; Wolf, 1978).

Several limitations follow from the pilot design. First, the study involved a single dyad, so the reliability and generality of the observed patterns require systematic replication across dyads, participants, and settings.

Second, programmed randomness, although conceptually appropriate, can obscure functional relations in small datasets. The unequal distribution of adverse events illustrates this problem: Player 1’s early exposure may have depressed performance independently of allocation skill, so between-player differences in Condition A reflect both strategy and event history. Future studies should balance, yoke, constrain, or explicitly model event exposure. Predetermining the card sequence was not used here because the tabletop game relies on draws from a shuffled common deck, and doing so would alter this game mechanic.

Third, the dependent measures were primarily in-game. Generalization to everyday time-management behavior should be assessed directly through external measures, observations across settings, and follow-up assessments rather than inferred from gameplay (Stokes & Baer, 1977).

Fourth, the study did not use a formal interobserver agreement protocol. One author extracted the measures, and the second verified them against video timestamps and final board states. This procedure could detect extraction errors but did not constitute independent double coding under a predefined agreement protocol. No quantitative agreement statistic could therefore be calculated, and coder-related error cannot be fully excluded.

Finally, the unstructured and unrecorded debriefing could not be analyzed as qualitative evidence. The proposed self-monitoring function of the game therefore remains theoretical. Future studies should use structured interviews or questionnaires, systematic qualitative analysis, and separate assessments of the social significance of the goals, the acceptability of the procedures, and the importance of the outcomes (Wolf, 1978).

Another limitation is the single fixed alternating sequence. The procedural similarity between Conditions A and B was intentional: the same board, task strips, temporal structure, and core response requirements were retained to minimize differences in rule learning and cognitive load and to preserve continuity across closely related game contexts. This choice was consistent with the educational rationale of supporting possible generalization of planning and allocation repertoires, although generalization was not assessed.

Within the alternating treatments design, however, this overlap created a potential carryover threat. Condition B did not function as a washout period or an uncontaminated baseline after exposure to Condition A. Four of the six B sessions immediately followed A, so response patterns, rules, or expectations developed in A may have generalized temporarily to B and reduced separation between conditions. Carryover in the opposite direction is also possible because five of the six A sessions followed B. Exposure to largely stochastic outcomes may have reduced the perceived utility of strategic responding in A, whereas the contrast between readily discriminable conditions may instead have increased sensitivity to the contingencies upon returning to A. The reduction in planning duration across both conditions also suggests a general familiarity or practice effect.

These alternatives cannot be distinguished because one dyad completed one fixed sequence with only one AA and one BB transition. The visual patterns should therefore be interpreted as descriptive contrasts under alternating exposure, not as evidence of an uncontaminated causal effect of Condition A. Future studies should use multiple dyads, counterbalanced or rotated sequences, balanced first-order transitions, and analyses that account explicitly for the immediately preceding condition.

These limitations suggest several next steps. Future studies should replicate the evaluation across multiple dyads, control event exposure through yoked or constrained randomization, and use independent coders with an appropriate reliability index. Component analyses could isolate the effects of the weekly review, escalating stress costs, and intermittent energy delivery. Educational impact should be assessed beyond gameplay through self-monitoring diaries, objective performance measures where feasible, standardized measures, and maintenance probes. Until such external and follow-up measures are available, claims about educational impact should remain limited to in-game performance.

Overall, the most conservative conclusion is that the design successfully produces behaviorally meaningful variability in performance under contingent versus noncontingent arrangements. The pilot also underscores a practical and methodological point for future evaluations: when chance is part of the model (as it should be for ecological validity), designs should explicitly manage or model the distribution of adverse events, because “luck” can meaningfully shape short-run outcomes, particularly in small samples. One practical option is a digital version that preserves stochasticity while allowing tighter control over the frequency and timing of adverse events. A remote computerized implementation would also reduce the logistical burden of alternating treatments designs, which typically require multiple sessions.

## 8. Conclusions

Regarding O1, the study provides a technological and behavior-analytic description of Steady State and its embedded measures. The game translates contingent reinforcement, aversive stimulation through increased response effort, intermittent schedules, differential reinforcement, and structured self-monitoring into a tabletop environment in which planning, task allocation, postponement, stress accumulation, and resource management can be observed repeatedly. Its compound consequence system includes intermittent delivery of energy units following task progress, consistent delivery of energy units contingent on task completion, and a weekly bonus contingent on balanced engagement across life domains (Ferster & Skinner, 1957; Skinner, 1953).

Regarding O2, the demonstrative pilot indicates that Steady State can generate measurable and behaviorally interpretable in-game variability. For RQ1, the embedded measures captured session-level variation in planning duration, task postponement, energy allocation, stress accumulation, and total session duration. For RQ2, visual inspection suggested greater between-player differentiation and a closer relation between individual allocation choices and performance in Condition A, whereas performance in Condition B was more strongly constrained by stochastic resource availability. These contrasts remain descriptive because the conditions also differed in rule complexity, effective strategy space, the functional relevance of player decisions, and session duration.

The pilot also showed that stochastic events influenced performance under the contingent arrangement. Adverse events altered short-term outcomes and interacted with allocation patterns, particularly in this single-dyad dataset. Player 1’s early sessions illustrate this interaction, with a high concentration of adverse events occurring alongside less efficient allocation and poorer outcomes. This is consistent with the game’s aim of modeling unpredictable disruptions, but it also shows that future studies should control, balance, or explicitly model adverse-event exposure when evaluating programmed contingencies and strategy development.

The evidence remains preliminary because of the single-dyad sample and variability introduced by random events. Nevertheless, the pilot indicates descriptively that Steady State can generate measurable and behaviorally interpretable in-game variability under contingent and largely noncontingent arrangements. The immediate priorities are systematic replication across additional dyads, stronger control or explicit modeling of event exposure, and direct assessment of generalization beyond gameplay (Stokes & Baer, 1977). If supported by future data, Steady State may function both as an educational resource for teaching self-management skills and as a research platform for studying allocation, persistence, and coping under realistic constraints and unpredictable disruptions.

As a final note, based on the pilot results, future full-scale studies should consider recruiting individuals who self-report time-management difficulties, because the game is most relevant when there is demonstrable room for improvement in scheduling, planning, and self-regulation repertoires. Participants with well-developed skills may show limited change, as illustrated by Player 2’s relatively stable performance. To reduce ceiling effects, future studies could include a brief self-report checklist and a brief baseline planning task.

## Figures and Tables

**Table 1:** Design logic of Steady State: game mechanics, behavior-analytic functions, and target in-game behaviors.

Game Mechanic	Behavior-Analytic Function	Target In-Game Behavior
Task strips	Discriminative stimuli and task demands	Task selection, sequencing, completion
Energy units	Putative conditioned reinforcers and response resources	Spending, saving, and allocating energy
Stress units	Increased response effort and aversive task demands	Avoiding overload and weekend spillover
Event cards	Programmed perturbations and shifts in establishing operations	Reallocation after disruption
Energy cards	Intermittent delivery of putative reinforcers following task progress	Continued engagement with tasks
Weekly review	Self-monitoring prompt and feedback opportunity	Reflection and rule formulation
Weekly balance bonus	Differential reinforcement for balanced allocation	Completing at least one task in each domain
Scoring rules	Delayed aggregate performance outcome	Cumulative planning across the session

**Table 2:** Morford et al.’s six “Characteristics of game-playing” matched to Steady State characteristics.

Characteristics of Game-Playing	Definition	Implementation in Steady State
Direct impact on the game outcome and results	A player’s behavior directly alters the characteristics of the outcome or the process of obtaining the outcome.	Task selection, energy allocation, and sequencing directly change session score and access to later options.
Clear goals and/or end conditions	A player is able to verbally specify a goal within a game or the conditions under which the game ends.	Fixed session length (32 turns), explicit scoring, and weekly bonuses specify end conditions and sub-goals.
Rules and barriers	A player’s behavior is restricted by rules (verbal behavior) or by physical barriers.	Stress costs, category requirements, and event constraints restrict response options.
Probabilistic outcome	The topography of the game outcome or process towards the outcome is probabilistic.	Random event/energy draws and variable task durations make outcomes and progress probabilistic.
Development of strategies and heuristics	A player can verbally evaluate the state of the game, and successive games played allows for the development of increasingly complex strategies (nonverbal) and heuristics (verbal) that may alter the probabilities of different outcomes.	Weekly reviews prompt verbal self-instructions and planning; repeated play supports emergent strategies (e.g., batching, front-loading).
Noncoerced Initiation	A player’s initiation and termination of the game occurs in the absence of coercion.	Participation is voluntary; players may start/stop sessions without social or instructional penalties.

**Table 3:** Task-category balance by session for Players 1 and 2 in Condition A, expressed as the proportion of weeks with at least one completed task in each category.

	Session 1	Session 2	Session 3	Session 4	Session 5	Session 6
**Player 1**	0.75	1	0.50	1	1	1
**Player 2**	0.75	1	1	1	1	1

**Table 4:** Distribution of adverse-event cards and cards drawn across Condition A sessions for Player 1.

Session	Unexpected	Extra Work	Illness	Delegation (Received)	Adverse Events/Cards Drawn
1	2	2	0	1	5/26 (19%)
2	2	2	2	0	6/25 (24%)
3	1	1	1	1	4/26 (15%)
4	1	0	2	2	5/27 (18%)
5	2	0	1	0	3/25 (12%)
6	0	0	2	1	3/28 (11%)

**Table 5:** Distribution of adverse-event cards and cards drawn across Condition A sessions for Player 2.

Session	Unexpected	Extra Work	Illness	Delegation (Received)	Adverse Events/Cards Drawn
1	0	0	1	0	1/27 (4%)
2	0	0	0	1	1/28 (4%)
3	0	1	1	0	2/28 (7%)
4	0	2	0	0	2/28 (7%)
5	0	2	0	2	4/28 (14%)
6	1	2	0	0	3/26 (11%)

**Table 6:** Results summary for Player 1.

Variable	Condition A	Condition B	Interpretation
Planning duration (seconds)	262	190	Longer planning in A reflects higher strategy demands
Delays (task-day shifts, mean cumulative count)	25.17	4.83	Greater sensitivity to the interaction between strategy and adverse events in Condition A
Unused energy (average)	2	0.5	Negligible resource surplus in Condition A possibly reflecting a inefficient energy management
Stress units (average)	1.67	2.33	Primarily determined by stochastic coin outcomes in Condition B; also elevated in Condition A
Category balance (mean rate)	0.88	N/A	Only meaningful in A

**Table 7:** Results summary for Player 2.

Variable	Condition A	Condition B	Interpretation
Planning duration (seconds)	271	153	Longer planning in A reflects higher strategy demands
Delays (task-day shifts, mean cumulative count)	1.83	4.33	Higher in Condition B, consistent with chance-determined variation and low postponement in Condition A
Unused energy (average)	11.3	2.5	Resource surplus in Condition A possibly reflecting efficient accumulation and buffering
Stress units (average)	0.83	1.83	Primarily determined by stochastic coin outcomes in Condition B
Category balance (mean rate)	0.96	N/A	Only meaningful in A

## Data Availability

The original contributions presented in this study are included in the article. Further inquiries can be directed to the corresponding author.
